# How to use near real-time health indicators to support decision-making during a heat wave: the example of the French heat wave warning system

**DOI:** 10.1371/4f83ebf72317d

**Published:** 2012-07-16

**Authors:** Mathilde Pascal, Karine Laaidi, Vérène Wagner, Aymeric Bun Ung, Sabira Smaili, Anne Fouillet, Céline Caserio-Schönemann, Pascal Beaudeau

**Affiliations:** Institut de Veille Sanitaire, Département Santé Environnement, Saint Maurice, France; Institut de Veille Sanitaire, Département Santé Environnement, Saint Maurice, France; Institut de Veille Sanitaire, Département Santé Environnement, Saint Maurice, France; Institut de Veille Sanitaire, Département Santé Environnement, Saint Maurice, France; Institut de Veille Sanitaire, Département Santé Environnement, Saint Maurice, France; Institut de Veille Sanitaire, Département de Coordination des Alertes et des Régions, Saint Maurice, France; Institut de veille sanitaire, Département de coordination des alertes et des régions; Institut de Veille Sanitaire, Département Santé Environnement, Saint Maurice, France

## Abstract

Introduction The French warning system for heat waves is based on meteorological forecasts. Near real-time health indicators are used to support decision-making, e.g. to extend the warning period, or to choose the most appropriate preventive measures. They must be analysed rapidly to provide decision-makers useful and in-time information. The objective of the study was to evaluate such health indicators. Methods A literature review identified a range of possible mortality and morbidity indicators. A reduced number were selected, based on several criteria including sensitivity to heat, reactivity, representativity and data quality. Two methods were proposed to identify indicator-based statistical alarms: historical limits or control charts, depending on data availability. The use of the indicators was examined using the 2006 and 2009 heat waves. Results Out of 25 possible indicators, 5 were selected: total mortality, total emergency calls, total emergency visits, emergency visits for people aged 75 and over and emergency visits for causes linked to heat. In 2006 and 2009, no clear increases were observed during the heat waves. The analyses of real-time health indicators showed there was no need to modify warning proposals based on meteorological parameters. Discussion These findings suggest that forecasted temperatures can be used to anticipate heat waves and promote preventive actions. Health indicators may not be needed to issue a heat wave alert, but daily surveillance of health indicators may be useful for decision-makers to adapt prevention measures.

## Introduction

The summer of 2003 in Europe was a sharp reminder that extreme temperatures remain a considerable danger for developed countries. In metropolitan France, nearly 15,000 deaths were recorded between the 1^st ^and the 20^th^of August 2003. The most vulnerable populations were the elderly, those suffering from chronic diseases, confined to bed, living alone or in social isolation, and outdoor workers^1^ ^2^ ^3^ . Since 2004, preventive actions targeting different vulnerable populations (the elderly, outdoor workers, sportsmen…) and health professionals have been implemented each summer to reduce the health impact related to heat. These actions are reinforced in case of a heat wave alert.

For the sake of clarity, in the following we have used the term “warning proposal” to refer to a heat wave warning based on forecasted meteorological data, the word “alarm” to refer to a public health alarm based on the observed health indicators, and the world “alert” to refer to the final decision taken by the prefect.

The heat wave warning system covers the 96 metropolitan French departements^4^ (a “department” in France is an administrative district) and runs from the 1^st ^June to the 31^st^ of August. Each day, forecasted meteorological data are analyzed by the weather services (Météo-France www.meteo.fr) and the French Institute for Public Health Surveillance (InVS). A warning proposal may be issued when both the 3-day averaged minimum and maximum forecasted temperatures have a high probability of exceeding predefined local thresholds. Additional information may be used to modify the proposal in order to allow greater adaptability to local climatic and human situations. This includes the intensity of the heat wave, relative humidity, likelihood of thunderstorms, peaks of air pollution, mass gatherings of people etc., and is assessed qualitatively. Warning proposals are transmitted by the InVS to the Ministry of Health, which in turn transmits the information to the prefects of the concerned departments. Information about the upcoming heat wave is communicated to the media and the general population by Météo-France through a vigilance map. The final decision of actually triggering the alert and therefore implementing prevention measures is the responsibility of the departmental prefects, who report directly to the authority of the Minister of the Interior and are in charge of the local emergency preparedness and response plans. Therefore, a warning proposal must contain enough information to motivate the prefects to issue the alert. In the subsequent days, updates provided to the prefect must clearly state when an alert should be ended.

During an alert, the monitoring of the health situation in the departments impacted by the heat wave can provide useful information for decision-makers. For example, real-time health indicators may identify a larger impact than expected, and therefore help orientate or reinforce preventive actions. They may also help in deciding when to end the alert. Such monitoring has been organised by the InVS since 2004, using daily collection of mortality and morbidity data for each department. With access to a growing number of available indicators through the development of a national syndromic surveillance system called SurSaUD®^5^ ^6^ ^7^ , and after several years of feedback and evaluation of the use of these indicators, we appraised a selection of indicators and methods that could be used to support decision making within the time and efficiency constraints of the existing warning system.

## Methods

**Definition and selection of the indicators.** In the framework of the heat wave prevention plan, we conceived the health indicators as decision-support tools which enable rapid action. The accurate retrospective description of the health impacts of the heat wave is addressed elsewhere 8 .

First, we identified possible health indicators associated with heat exposure, identified by reviewing published (using Medline), grey and unpublished literature (obtained from our partners). Then, we defined several criteria to select the most relevant indicators, using more general assessments of health indicator quality^9^ ;

- Reactivity, i.e. the lag between exposure to heat and a variation in the indicator trend;

- Delay in obtaining data;

- Existence of a clear and common definition used by all data providers

- Possibility that the observed variations are caused by factors other than heat (confounding factors);

- Comparability of the indicator between different geographic areas and time periods;

- Data quality and reliability (quality insurance, consistency with data collected by other sources);

- Interpretability: possibility of drawing meaningful conclusions from the indicators, in order to promote health actions.

Information to ensure each criterion was met was documented based on the literature review, on a review of the existing surveillance systems used to measure the possible indicators in France, and on expert judgment.

**Statistical analysis.** Once the indicators were selected, we chose two statistical methods to analyse them during alerts. These statistical methods needed to be able to identify an unusual excess of cases, computed as the difference between the numbers of observed and expected cases. They had to rely on robust statistical hypotheses, and be easy to perform automatically on a daily basis. Considering the strong limitation due to anteriority of data available, we selected the historical limits method when at least two years of data were available, and a method based on control charts when less than two years of data were available.

The historical limits method is performed by the US Centres for Disease Control and Prevention for their “Morbidity and Mortality Weekly Reports”. This method computes the ratio of the observed value on day d of year n to the mean of the observed values at days d-k, d-k+1,…,d-1, d, d+1,…,d+k of years n-1 to n-q. A statistical alarm is triggered when this ratio exceeds 1+2σ/μ, where μ is the mean and σ is the standard deviation computed from all the observations 10 . For our purposes, the observed values on day d of year n were compared to the mean of the observed values for the same day of the same week, during the previous and following weeks, for each year of the historical data.

The control chart method compares the observation of day d with a threshold calculated from previous data. This threshold was set as the mean of the observed values for the same day during the 3 previous weeks, with the addition of 3 standard deviations of these observations 11 .

For both methods, observations have to follow a Gaussian distribution and be independent. Long-term trends are not taken into account. We did not define a “minimum number of cases” required to perform these methods, although we supposed, based on experience, that it would be difficult to detect an increase when less than 20 cases per day occurred.

**Use of the health indicators.** During an alert, indicators are analysed daily in each concerned InVS’s regional office using one of the statistical methods described above. A statistical alarm on the health indicators initiates an investigation by local epidemiologists in close relation with data providers (hospitals, practitioners…) before being considered a valid alarm. If the alarm is validated, an analysis of the health situation is then transmitted to the health authorities. It may be used to modify existing preventive actions, or to keep a warning in place after temperatures decrease.

Situations where no statistical alarm is triggered but when an indicators shows a sustained increase in activity (several days above usual values), or when several indicators show increasing trends in the same period are also considered for further investigation.

The whole procedure relies on the local expertise of epidemiologists, especially for morbidity indicators which are highly sensitive to external events that may affect the numbers of those seeking care, such as tourist flow or seasonal departures, depending on the region.

Simulations on how the chosen health indicators would react were performed for heat waves observed in 2006 and 2009. We focused on departments which are heterogeneous in terms of population and data availability: Bouches-du-Rhône, Ardèche, Drôme, Vaucluse, Tarn, Haute-Garonne, Tarn-et-Garonne, Rhône,Paris, Hérault and Indre-et-Loire.

## Results

**Selection of the indicators.** Based on the literature review, we primarily identified 11 mortality indicators (Table 1) and 14 morbidity outcomes (Table 2) associated with heat exposure.

Mortality indicators included total mortality, mortality by age group, by sex and by cause. As each cause of mortality may be monitored for different age groups and sex, the number of potential indicators can become very large. The literature is consistent about a higher risk of mortality in older age groups 12 13 , whereas results are less clear for the youngest age groups 13 14 . Similarly, a higher risk has been observed in women in some^13^but not all studies^14^ . Therefore, we did not distinguish age groups (except over 75 years of age) and sex. Indicators are qualitatively assessed in Table 1.

For total mortality, neoplasms and heat-related mortality, the lag between exposure and the increase in mortality seen in previous studies is usually between 0 and 4 days, with the main peak occurring in less than 48 hours^12^ ^13^ ^14^ ^15^ ^16^ ^17^. On the contrary, a lag of 0-15 days has been observed for cardiovascular^18^ ^19^ ^20^ ^21^ and respiratory mortality^19^ ^20^ ^22^ , limiting the reactivity of these indicators.

The main limitation regarding total mortality is the delay in obtaining the data, which limits the usefulness of following mortality for decision-making purposes during short heat waves. Existing surveillance systems in France include two mortality sources: the total mortality records from the French National Institute for Statistics and Economic Studies (data from 3 000 cities were collected in 2011, representing 80% of national mortality), and mortality by cause, reported by physicians through a system of e-certification of death (currently representing 5% of the national mortality)^5^ ^6^ ^7^ . Total mortality indicators are available on average 3 to 4 days after deaths, but 7 days are required to obtain validated data. Data on in-hospital deaths can be obtained with a lag of 1-2 days. Mortality by cause is available on the day of the death only for a very small percentage of all deaths.

Overall, data quality was considered very good, except for heat-related mortality, which may have been associated with declaration biases (mainly under-reporting).

**Table 1 – Possible mortality indicators:** **** Very good, *** Good, ** Poor, * Very poor

**Indicator**	**Reactivity**	**Delay**	**Definition**	**Confounding factors**	**Comparability**	**Data quality**	**Interpretability**
**Total mortality**	***	*	****	***	****	****	****
**Total mortality by age group**	***	*	****	**	****	****	***
**Total mortality by sex**	***	*	****	**	****	****	**
**Neoplasms**	**	**	****	**	*	****	**
**Cardiovascular causes**	*	**	****	**	*	****	**
**Respiratory causes**	*	**	****	**	*	****	**
**Heat-related causes**	***	**	**	****	*	**	****
**In-hospital deaths**	***	****	****	**	***	****	**

The relevance of morbidity indicators is assessed in Table 2. In the literature, the number of hospital emergency visits has been found to usually moderately increase during heat waves 23 24 for all age groups: above 75 years old 7 , above 65 years old^25^, and even for younger age groups^25^. Reported causes have been dehydration, hyperthermia and heat stroke 7 25 26, renal diseases 25 26 , and cardiovascular and cerebrovascular diseases 25 27 , visits linked to alcohol or drugs consumption, and violence28, as well as hyponatremia in elderly people 7. The peak has usually been observed 0 to 1 day after the peak in temperature 29 30 31. An increase in emergency calls to 911 has been documented in the United States^32^, while an increase in emergency calls to NHS direct has been observed in the UK 33. In 2003,France recorded increases of emergency calls to GPs, hospital emergency visits, activity of emergency ambulance services (SAMU) and fire brigades.

In France, total hospital emergency visits by age group are available in each department with a lag of 1 day. In addition, emergency visits by cause are available through OSCOUR®, an emergency departments (ED) network (about 660 ED were involved in 2011, representing more than 50% of the French hospitals) 5 6 7 . However, as the recruitment of the hospitals in this network is based on voluntary-membership, some regions are only partially covered and two (Auvergne and the island of Corsica) are not yet covered at all in the French metropolitan area.

Regarding emergency calls, possible indicators are calls to GP’s emergency associations (SOS-Médecins), the activity of the emergency ambulance services (SAMU) and the activity of the fire brigades (Table 2). The SurSaUD® database gathers OSCOUR® ED data and SOS-Médecins data, including the number of emergency calls, administrative and demographic information (age, sex, zip code of residence etc.), the chief health complaint, and for some associations, the diagnosis performed by the GP during a home visit following an emergency call. Data are available for 59 SOS-Médecins associations out of 62, located in the main cities. SOS-Médecins data from the SurSaUD® database are coded with quite good quality using two different thesauruses. In 2011 for example, percentages of chief complaints and diagnosis coded according to the SOS-Médecins thesauruses were approximately 99% and 63% respectively^34^ .

Other available indicators are the total number of interventions performed by the SAMU and, in some regions, the total number of interventions delegated by the SAMU to the fire brigades. However, differences in organization and definitions between departments limit the interregional comparability of these indicators.

Finally, based on their reactivity, representativity and data quality, we selected one mortality indicator and four morbidity indicators to be investigated on a systematic basis during a heat wave as follows:

- total mortality;

- emergency visits (total)

- emergency visits (over 75 years-old)

- emergency visits for heat-related causes (hyperthermia, hyponatremia and dehydration);

- SOS-Médecins calls.

It is of course possible to investigate additional indicators (by age group, by cause), depending on data availability. In-hospital deaths cannot be used to monitor the situations of those who do not use health services, but only act as an indicator of the gravity of the impact of the heat wave, thereby complementing morbidity indicators.

**Table 2 – Possible morbidity indicators:** **** Very good, *** Good, ** Poor, * Very poor

**Indicator**	**Reactivity**	**Delay**	**Definition**	**Confounding factors**	**Comparability**	**Data quality**	**Interpretability**
**SAMU and fire brigades**	****	****	****	*	*	**	***
**Total emergency visits**	****	****	****	*	**	***	****
**Visits by age group**	****	****	****	*	**	***	***
**Visits for hyperthermia**	****	****	***	****	*	**	***
**Visits for dehydration**	****	****	***	***	*	**	**
**Visits for hyponatremia**	****	****	***	****	*	***	***
**Visits for cardiovascular and respiratory causes**	**	****	***	*	*	***	**
**Visits for kidney diseases**	****	****	***	*	*	***	**
**Visits for drug abuse, violence**	****	****	***	*	*	***	**
**Hospital admissions following emergency visits**	****	****	****	*	*	***	**
**GP emergency visits (SOS Médecins)**	****	****	***	*	**	***	

**Illustration of the use of the selected indicators: 2006 and 2009 heat waves.** Two main heat waves were observed in 2006 and 2009. Table 3 presents a summary of heat waves characteristics and of the available health indicators for 2006 and 2009 in the impacted departments.

In July 2006, heat wave warning proposals were issued for several departments, lasting up to 31 days in the Bouches-du-Rhône department. In all departments, the warning proposals were transformed into alerts by the prefects.

During that period, several statistical alarms were observed for total hospital emergency visits, hospital emergency visits for people over 75 years-old and mortality. Similar situations were observed in Hérault, Indre-et-Loire,Paris and Rhône: statistical alarms were observed during several consecutive days for the hospital emergency visits for people over 75 years-old. In Haute-Garonne, isolated statistical alarms were observed, but did not occur during or after the hottest periods.

In 2009, a first warning proposal was issued on the 16^th^ August for the Rhône, while a warning proposal with no subsequent alert was communicated for Drôme, Ardèche, Haute-Garonne, Tarn and Tarn-et-Garonne. Warning proposals were transformed into alerts for all these departments plus Vaucluse, issued on the 18^th^ August. The heat wave ended on the 20^th ^and 21^st^ August, depending on the department. In Vaucluse, no major health impact was observed, but four alarms were issued following analysis of health data - three of these when both methods were used and one using the historical limit method only. One alarm occurred on the 21^st^ August during the heat wave, the other three before the heat wave (11^th^, 12^th^, 15^th^ August, when night temperatures were already high) (Figure 1). As temperatures were just slightly below thresholds, taking into account the health information could have led to the warning proposal being issued between the 11^th^ and the 15^th^ August.

In Ardèche, Tarn, Tarn-et-Garonne and Drôme, isolated statistical alarms were observed on some days, but were not associated with and did not parallel warm temperatures. In Tarn and Tarn-et-Garonne, statistical alarms were also observed on the 21^st^ and 22^nd^ August, which may be a lagged effect of the heat episode.

The “hospital emergency visits for heat-related causes” indicator was available in Paris only. It increased consistently with temperatures, but the small number of cases (0 to 12 cases per day, compared with 1,200 visits in total) does not allow any statistical analysis. SOS-Médecins data were not available during these heat waves.

**Table 3 – Local meteorological indicators of heat waves (3-day average of temperatures) and daily number of cases for health indicators, during the 2006 and 2009 heat waves (mean [min-max]):** 

**French department**	**Year**	**3-day average of minimal temperatures**	**3-day average of maximal temperatures**	**Emergency visits**	**Emergency visits>75**	**Deaths**	**Emergency visits for heat-related causes**
**Ardèche**	2009	16.5 [10.6:19.6]	30.4 [22.5:37.0]	90 [59:116]	9[2:17]	4 [1:11]	
**Bouches-du-Rhône **	2006	20.6 [9.6:24.5]	31.2 [22.5:36]	1050 [775:1346]	101 [75:130]	35 [22:52]	_
**Drôme**	2009	17.8 [12.2:21.1]	30.5 [23.1:37.4]	137 [89:167]	13[4:23]	6 [1:12]	_
**Haute-Garonne**	2006	17.5[8.7:22]	28.9 [21.5:36.5]	313 [244:442]	32 [21:48]	5 [0:10]	1 [0:5]
**Hérault**	2006	19.9[10.3:24]	30.3[23.6:35]	210 [133:274]	16[7:27]	9 [3:17]	_
**Indre et Loire**	2006	14.9[6:20]	26.6 [19.0:35.2]	197 [154:241]	22[12:41]	5 [1:14]	_
**Paris** ****	2006	16.5 [7.9:21.8]	25.8 [17.8:35.2]	1000 [767:1273]	70[42:101]	40 [23:58]	3[0:13]
**Rhône**	2006	17.4[7.8:23]	28.2 [17.2:37]	270 [94:321]	23[12:41]	13 [5:23]	_
**Tarn** ****	2009	16.0[9.5:20.9]	29.9 [22.5:37.5]	87 [39:132]	12[4:23]	1[0:5]	_
**Tarn-et-Garonne**	2009	16.3 [10.6:20.3]	29.2 [23.2:35.9]	96[63:122]	11[3:24]	_	
**Vaucluse**	2006	18.9[10:22.6]	31.8 [20.7:37.6]	415 [305:523]	45[28:59]	7 [3:15]	_

**Evolution of hospital emergency visits in the Vaucluse department in August 2009, and alarm thresholds calculated using two methods: either historical limits or control charts.**
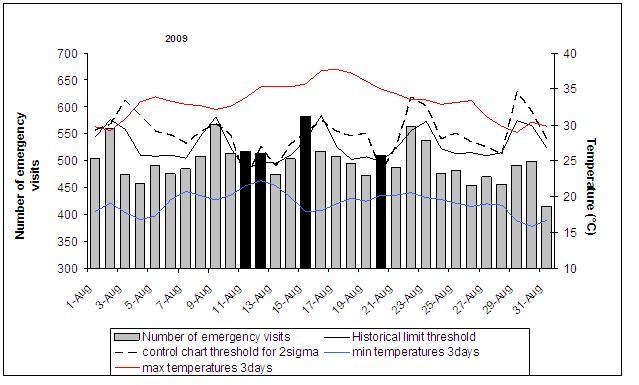
Black indicates days with a statistical alarm on the indicator

## Discussion

The heat warning system in France relies on meteorological forecasts and is able to anticipate dangerous heat waves from 24 to 72 hours in advance. It is included in a national management plan capable of setting up timely appropriate actions, thanks to early warnings. Warning thresholds have been set in order to anticipate a major public health impact and to implement pre-defined actions. During less intense heat waves, the media are used to relay appropriate information to the general population. The system is flexible enough to allow extensive discussion between health and meteorological services, and to modify the rules depending on the local situation.

These findings suggest that in France health indicators may not useful to issue a warning proposal. Indeed, since 2004, the decisions taken based on meteorological forecasts have not been modified by the subsequent analyses of real-time health indicators. No marked peaks in the health indicators have been observed during heat wave periods, except for emergency visits for heat-related causes, which is very sensitive to heat, even though representing very small numbers of daily cases. Nevertheless, health indicators would be fundamentally important when assessing the appropriateness of implemented prevention measures on the general population and the need for additional response services. They are also very useful as they reassure health authorities and give them objective data.

As in general, indicators must be interpreted quickly, we preferred to reduce the number to be considered during a heat wave warning and to harmonise the statistical methods used to analyse them between departments. Based on the literature review and data quality assessment, we considered that mortality, hospital emergency visits and emergency calls were indicators to be kept. Mortality indicators are limited by the delay in obtaining the data, while morbidity indicators are limited by data availability and quality. These latter are considered most attractive, as they are more reactive, and still allow life-saving interventions. However, it is possible that morbidity data could remain stable during a heat wave while mortality increases. For longer heat waves, mortality data can still be used to orientate the actions. Finally, when several heat waves are observed over the same summer in the same department, mortality data can be used to assess the vulnerability of the population and to qualitatively predict how they may react to a new episode. In an initial analysis, we decided to focus on all ages and on the older age group (>75 years old). Younger age groups, especially infants, were not selected as the small numbers render interpretation difficult. There is a need for methods to detect and interpret an increase in time-series with low number of daily cases.

Data availability and data quality remain the two main limits of this system. Data availability should improve in the coming years with the development of the Sursaud © network. Data quality is a key issue that should also improve as physicians get more familiar with the systems and its objectives. Feedback on the use of these data to the health professionals is therefore an asset, to improve their understanding of the health impacts during an heat wave, but also to promote data collection and transmission.

In addition to those indicators routinely used to support decision-making during heat waves, it is important to allow greater adaptability to local climatic and human situations. This is done through qualitatively investigating signals from several indicators that are not statistically analysed, such as emergency visits for infants or for cardiovascular diseases. Close connections with health professionals are also required to collect information which can be investigated later, for instance, asking about a possible connection with heat when physicians report an excess of specific pathologies.

The analysis of real-time indicators highlighted here is no substitute for future epidemiological investigation of the impact of temperature on morbidity. Such indicators are part of the decision-making puzzle and cannot be interpreted in a different context. The challenge therefore is to ensure clear communication of the meaning and limitations of these indicators, and to avoid misinterpretation by health professionals and decision-makers.

Bulletins have been developed by InVS regional offices and are used to communicate at the regional level to the stakeholders. Several models have been developed, which usually include a qualitative assessment of the evolution of the indicators (increase, decrease, stable)^36,35^ 35 36 37 38 .

A feedback on the health surveillance is also produced at the end of the summer 38 . At the national level, a summary of the main points of the regional bulletins transmitted daily to all the stakeholders of the systems during a warning.

These health indicators were chosen specifically for the follow-up of heat waves, in association with an analysis of meteorological forecasts, within a well-defined heat wave prevention plan. The method to select relevant indicators could be applied in other countries planning to use of health indicators within a heat prevention plan.

Health indicators could also been defined to follow-up other extreme weather events (storms, floods, forest fires….). Unlike heat waves, these events are characterized by a great variability of possible scenarios and means of exposure to health risks. Their health impacts are therefore more wide-ranging than the immediate fatalities and physical traumatisms. Epidemiological surveillance must therefore be flexible and reactive in order to adapt to event-specific scenarios and to implemented actions 39 . However, the criteria used to assess the quality of the indicators could also be applied.
